# Neonatal Vitamin D status and the risk of neonatal sepsis

**DOI:** 10.12669/pjms.35.2.342

**Published:** 2019

**Authors:** Abdurrahman Avar Ozdemir, Yakup Cag

**Affiliations:** 1 *Abdurrahman Avar Ozdemir Department of Pediatrics, Medicine Hospital, Biruni University, Istanbul, Turkey*; 2 *Yakup Cag Department of Pediatrics, Dr. Lutfi Kirdar Training and Research Hospital, Medical Sciences University, Istanbul, Turkey*

**Keywords:** Vitamin D, Sepsis, Neonate

## Abstract

**Objective:**

To evaluate the maternal and neonatal 25-hydroxyvitamin D [25(OH)D] levels and the effect of 25(OH)D levels on the development of neonatal sepsis.

**Methods:**

This prospective study was performed in the neonatal intensive care unit of Medicine Hospital/Biruni University between November 2017 and September 2018. Fifty one term infants with sepsis group and 56 term infants with control group were included in this study. Blood samples for whole blood count, CRP, Ca, P, ALP, 25(OH)D and culture were obtained from all neonates.

**Results:**

Mean vitamin D levels for the neonates and their mothers were found to be 12.4±8.5 ng/ml and 13±8.7 ng/ml, respectively. There was a significant correlation between maternal and newborn 25(OH)D levels (r=0.72, p<0.01). The number of the newborns with vitamin D deficiency was significantly higher in the sepsis group (n=31, 60.8%) than in the control group (n=30, 53.6%; p=0.00), corresponding to significantly lower levels of vitamin D in the sepsis group (11±5.5 ng/ml vs. 13.8±10.6 ng/ml; p=0.012). Similarly, maternal vitamin D levels was significantly lower in the sepsis group than in the control group (10.8±5.6 ng/ml *vs*. 14.9±10 ng/ml; p=0.001).

**Conclusion:**

Our findings suggest that there may be an association between vitamin D deficiency and neonatal sepsis

## INTRODUCTION

Vitamin D is a lipid-soluble prohormone produced in the human skin exposure to ultraviolet radiation. It plays an important role in calcium and phosphorus homeostasis.^1^,^2^ Although its deficiency is generally linked to the disorders of bone mineralization, there is some evidence suggesting that vitamin D also plays a role in the immune function.^3^,^4^ Vitamin D receptors are widely expressed in the epithelial tissue and cells of the immune system. 1,25-dihydroxyvitamin D (1,25(OH)2D), an active form of vitamin D, acts as an immune modulator to stimulates the innate immune system.^3^,^4^

In several studies of adults low vitamin D levels have been found to be associated with predisposition to sepsis.^5^,^6^ Similarly, in some studies of children, significant associations have been shown between vitamin D deficiency and respiratory tract infections and sepsis.^7^,^8^ However, there are limited data on the role of vitamin D in neonatal sepsis. Neonatal sepsis refers to bacteremia and associated clinical signs and symptoms occurring in the first month of life.^9^,^10^ It is among the leading causes of morbidity and mortality in newborns. As the immune system of newborns has not fully developed at birth, they are relatively immunocompromised, rendering them vulnerable to infections.^11^

In this prospective study, we aimed to evaluate the maternal and neonatal vitamin D status and the effect of vitamin D levels on the development of neonatal sepsis.

## METHODS

This prospective study was performed in the neonatal intensive care unit of Medicine Hospital/Biruni University between November 2017 and September 2018. The study protocol was approved by the ethics committee of university and informed written consent was obtained from all mothers.

Exclusion criteria for newborns included the presence of the following: congenital malformation or disease, metabolic disease, small for gestational age (SGA), prematurity, twin neonates, use of antibiotic therapy at admission, and age above 28 days. Exclusion criteria for mothers included malnutrition, metabolic or chronic disease, twin pregnancy, use of medications, chorioamnionitis, premature rupture of membrans, and age less than 20 years or above 40 years.

The study group comprised term newborns with laboratory and clinical findings of sepsis, which was further categorized as early onset (EOS; within ≤3 days of birth) or late onset (LOS; after three days of birth) neonatal sepsis. Demographic features including gestational age, gender, weight, mode of delivery, Apgar score, season and prenatal findings were recorded. Blood samples for the whole blood count, C-reactive protein (CRP), and culture were obtained from all neonates with suspected sepsis at admission and the hematologic parameters were evaluated according to the Manroe and Rodwell scoring systems.^12^,^13^ The neonates were categorized into 4 groups (highly probable sepsis; Group-I, probable sepsis; Group-II, possible sepsis; Group-III and no sepsis; Group-IV) according to the criteria defined by Gitto et al.^14^ The newborns in groups 1,2 and 3 were called as sepsis group and others with no signs of neonatal sepsis served as the control group, which consisted of infants diagnosed with transient tachypnea of the newborn (TTN) or neonatal hyperbilirubinemia.

Demographic features of the mothers such as age, gender, parity, socio-economic status, education level, and the factors associated with their vitamin D status (sun exposure, vitamin D intake, style of clothing and season) were recorded. Wearing traditional clothes covering the arms, legs and head was accepted a covered clothing style. Limited exposure to sunlight was considered to spend less than 30 minutes outside during the day. The mothers were also classified into three groups according to their daily vitamin D intake: none, insufficient (irregular or low-dose) and sufficient. In Turkey, a vitamin D supplementation program has been in operation, with daily provision of 1200 IU vitamin D to all pregnant women from the first trimester until six months after delivery.

Blood samples for calcium (Ca), phosphorus (P), alkaline phosphatase (ALP) and 25(OH)D were obtained from all participants at admission. The 25(OH)D levels were measured by an enzyme-linked fluorescent assay on the Architect i2000SR analyzer (Abbott Laboratories, US) and the Ca, P, ALP and CRP levels were measured using the photometry method on the Architect C8000 analyzer (Abbott Laboratories, U.S.A). Complete blood count measurements were performed by using th Cell-Dyn Ruby System (Abbott Laboratories, US). 25(OH) vitamin D levels were classified according to the guidelines of the Endocrine Society; as sufficient for >20 ng/ml (>50 nmol/l), insufficiency for 12-20 ng/ml (30-50 nmol/l), and deficiency for <12 ng/ml (30 nmol/l). The participants were classified into 3 group’s accordingly.^15^

Data were analyzed using the SPSS Statistics 20 program. Descriptive variables were expressed as mean, median, standard deviation, minimum, maximum and percentages. Between group differences were evaluated using Student’s-t test, Chi squared test, Mann-Whitney U test and correlations between quantitative data were analyzed by the Spearman’s correlation test. A *p* value of less than 0.05 was considered to be significant.

## RESULTS

This study included 107 term neonates (41 females, 66 males), of which 51 (47.7%) were in the sepsis group and 56 (52.3%) were in the control group. The mean gestational age was 38±1.3 weeks, and the mean birth weight was 3.338±482 g. There were no significant differences between the two groups with regard to gestation week, birth weight, type of delivery, and Apgar score. The number of male newborns was significantly higher in the sepsis group (p=0.01). The newborns in the sepsis group had significantly higher CRP levels (p=0.001) and significantly lower platelet counts (p=0.04) (Table-I).

**Table-I:** Clinical characteristics of the newborns with or without sepsis.

	Sepsis Group (n=51) (Mean±SD)	Control Group (n=56) (Mean±SD)	p
Gestational Age (week)	38.1±1.5	38.2±0.9	^1^0.74
***Gender***			
Female	11 (21.6%)	30 (53.6%)	^2^0.01*
Male	40 (78.4%)	26 (46.4%)	
Birth weight (g)	3346±574	3331±401	^1^0.43
***Delivery route***			
Normal	41 (80.4%)	36 (64.3%)	^2^0.06
Cesarean	10 (19.6%)	20 (35.7%)	
Apgar score min.1	8±1	8±1	^1^0.7
Apgar score min.5	9±1	9±1	^1^0.6
CRP (mg/L)	17.9±15	1.9±2.1	^1^0.001*
Leukocyte (/mm^3)^	16278±6782	17841±6999	^1^0.32
Platelet (/mm^3)^	224989±103104	266916±66060	^1^0.04**

* Statistically significant at 0.01,

** Statistically significant at 0.05;

1 : Student’s t-test

2 : Chi-squared test

The mothers of the newborns in the two groups did not differ significantly with regard to age, number of births, socio-economic level, daily sun exposure, and style of clothing. The number of the mothers who took vitamin D during pregnancy was higher in the control group than in the sepsis group (p=0.02). Based on the seasonal distribution of deliveries, the number or winter deliveries was significantly higher in the sepsis group (p=0.016) (Table-II).

**Table-II:** Maternal characteristics of the newborns.

	Sepsis Group (n=51) (Mean±SD)	Control Group (n=56) (Mean±SD)	p
Age (year)	30±6	30±4	^1^0.93
***Parity***			
1	23 (45.1%)	21 (37.5%)	^2^0.69
2	18 (35.3%)	21 (37.5%)	
≥3	10 (19.6%)	14 (25%)	
***Socio-economic status***			
Low	10 (19.6%)	9 (16.1%)	^2^0.77
Moderate	38 (74.5%)	42 (75%)	
High	3 (5.9%)	5 (8.9%)	
***Education level***			
Primary	12 (23.5%)	8 (14.3%)	^2^0.41
Secondary	25 (49%)	28 (50%)	
High	14 (27.5%)	20 (35.7%)	
***Daily vitamin D intake***			
None	16 (31.4%)	16 (28.6%)	^2^0.02
Insufficient	27 (52.9%)	19 (33.9%)	
Sufficient	8 (15.7%)	21 (37.5%)	
***Daily sunlight exposure***			
Yes	12 (23.5%)	13 (23.2%)	^2^0.06
No	39 (76.5%)	43 (76.8%)	
***Clothing style***			
Covered	35 (68.6%)	33 (58.9%)	^2^0.3
Uncovered	16 (31.4%)	23 (41.1%)	
***Season***			
Winter	27 (52.9%)	15 (26.8%)	^2^0.016*
Spring	11 (21.6%)	17 (30.4%)	
Summer	5 (9.8%)	16 (28.6%)	
Fall	8 (15.7%)	8 (14.3%)	

* Statistically significant at 0.05;

1 : Student’s t-test

2 : Chi-squared test

Overall, the mean 25(OH)D level was 12.4±8.5 ng/ml in the infants and 13±8.7 ng/ml in the mothers, with a significant correlation between maternal and infant 25(OH)D levels (r=0.72, p<0.01). Vitamin D deficiency was detected in 61 newborns (57%) and in 55 mothers (51.4%). The number of the newborns with vitamin D deficiency was significantly higher in the sepsis group (n=31, 60.8%) than in the control group (n=30, 53.6%; p=0.00), corresponding to significantly lower levels of vitamin D in the sepsis group (11±5.5 ng/ml *vs*. 13.8±10.6 ng/ml; p=0.012). Similarly, maternal vitamin D levels was significantly lower in the sepsis group than in the control group (10.8±5.6 ng/ml *vs*. 14.9±10 ng/ml; p=0.001). Other laboratory newborn and maternal markers including Ca, P and ALP were similar in the two groups (Table-III, IV).

**Table-III:** Laboratory findings of the newborns.

	Sepsis Group	Control Group	p
Number of infants (n, %)	51 (47.7%)	56 (52.3%)	
25 (OH) D (ng/mL) (mean±SD)	11.0 ± 5.5	13.8 ± 10.6	^1^0.012*
Ca (mg/dL) (mean±SD)	8.7 ± 1.2	8.9 ± 0.7	^2^0.36
P (mg/dL) (mean±SD)	5.9 ± 0.9	5.9 ± 0.9	^2^0.74
ALP (U/L) (mean±SD)	163 ± 58	162 ± 48	^2^0.91

25(OH)D: 25-hydroxyvitamin D; Ca: Calcium; P: Phosphorus; ALP: alkaline phosphatase.

* Statistically significant at 0.05;

1 : Mann-Whitney U test,

2 : Student’s t test

**Table-IV:** Laboratory findings of the mothers.

	Sepsis Group	Control Group	p
Number of mothers (n, %)	51 (47.7%)	56 (52.3%)	
25 (OH) D (ng/mL) (mean±SD)	10.8 ± 5.6	14.9±10	^1^<0.001*
Ca (mg/dL) (mean±SD)	9.0 ± 0.5	8.8 ± 0.4	^2^0.10
P (mg/dL) (mean±SD)	3.9 ± 0.5	4.3 ± 1.0	^2^0.42
ALP (U/L) (mean±SD)	115 ± 38	116 ± 30	^2^0.91

25(OH)D: 25-hydroxyvitamin D; Ca: Calcium; P: Phosphorus; ALP: alkaline phosphatase.

* Statistically significant at 0.01;

1 : Mann-Whitney U test,

2 :Student’s t test

Of 51 newborns with sepsis, 39 (76.5%) and 12 (23.5%) newborns were identified as having EOS and LOS, respectively. The newborns with EOS (10.4±5.7 ng/ml) had lower vitamin D levels than those with LOS (12.8±4.3 ng/ml), but the difference did not reach statistical significance (p=0.075). However, the mean vitamin D level of the newborns with EOS significantly differed from that of the control group (p=0.02), which was not the case for the newborns with LOS (p=0.7). The mean vitamin D levels were similar among patients with positive (n=11; 10.4±5.4 ng/ml) and negative (n=40; 11±5.3 ng/ml) blood cultures and the newborns in the control group (p=0.7, p=0.2).

## DISCUSSION

Demonstration of vitamin D receptors in immune system cells other than the extraskeletal system has drawn attention to the effects of vitamin D on the immune system, especially in association with sepsis, but the underlying pathological process has yet to be clarified. Vitamin D receptors are found in CD4 and CD8 T cells, B cells, neutrophils, macrophages and dendritic cells that play a role in the innate and adaptive immune response. Studies have shown that vitamin D has a suppressing effect on proliferation and antibody production of T and B cells as well as on the immune response of monocytes and dendritic cells. These findings suggest that vitamin D may play a role in autoimmunity.^4^,^5^,^16^ In studies on the innate immune system, vitamin D has been found to activate Toll-like receptors (TLR), which in turn induce the production of peptides such as cathelicidin and beta-defensin that have antimicrobial effects on bacteria, viruses and fungi.^5^,^6^ Early in vitro studies showed that Vitamin D had an inhibitory effect on *Staphylococcus aureus*, *Streptococcus pyogenes*, *Klebsiella pneumoniae*, and *Escherichia coli*, but its effect on fungal and parasitic infections remains unclear.^4^,^17^

In a meta-analysis, vitamin D deficiency was found to be a risk factor for infection and sepsis, resulting in increased mortality in adult patients in intensive care unit.^18^ Madden et al. assessed admission vitamin D levels in critically ill children and found a high rate of vitamin D deficiency (40%), which was also associated with increased disease severity at admission.^19^ In contrast, Ponnarmeni et al. reported no association between low vitamin D levels and increased severity of illness in critically ill children with sepsis.^20^

There are few studies investigating the relationship between sepsis and vitamin D in the neonatal period. Cetinkaya et al. reported a mean vitamin D level of 8.6 ng/ml in term newborns with EOS, as compared with 19 ng/ml in the control group.^21^ Similarly, Kanth et al. found significantly lower vitamin D levels in newborns with EOS than in the control group (14.6 ng/ml *vs*. 26.4 ng/ml).^22^ Gamal et al. found not only significantly lower vitamin D levels in newborns with EOS as compared with controls, but also significant inverse correlations between vitamin D levels and all sepsis markers.^23^ Of note, all these studies reported a positive correlation between maternal and infant vitamin D levels. Our findings are consistent with the aforementioned studies of maternal and infant vitamin D levels in the context of newborn sepsis, but with a clear distinction. Previous studies assessed vitamin D levels only in newborns with EOS, whereas our study included all newborns with EOS and LOS. Although vitamin D levels of the newborns with EOS and LOS did not differ significantly, only newborns with EOS had significantly lower levels of vitamin D when compared with the controls. This finding may provide further insight into the course of newborn vitamin D levels in relation to EOS and LOS. Identification of significantly lower vitamin D levels in newborns with EOS and the correlation between maternal and newborn vitamin D levels might suggest that low vitamin D levels are likely to predispose newborns to EOS, whereas LOS is more likely to result from nosocomial and environmental factors other than vitamin D levels.

Among demographic features and birth-related factors, only male gender was significantly predominant among newborns with sepsis. It is well-known that male sex is a risk factor for sepsis in the neonatal period, which is thought to be associated with the suppressive effects of androgens on the immune system.^24^ As expected, compared with the control group, the newborns in the sepsis group had significantly higher CRP levels and significantly lower platelet counts.

As the mothers are the main source of neonatal vitamin D levels, its insufficiency or deficiency affects not only the mothers but also their babies. The Institute of Medicine emphasizes the need for at least 400-600 IU/day of vitamin D intake for pregnant women. In Turkey, a vitamin D supplementation program has been implemented, aiming to provide 1200 IU/day vitamin D to all pregnant women. Before the introduction of the supplementation program, high rates of vitamin D deficiency had been reported among pregnant women, ranging between 50% and 94%.^25^ In the current study, vitamin D deficiency was documented in 51.4% of the mothers and the rate of women who had regularly received vitamin D supplementation throughout pregnancy was as low as 27%. Comparisons with respect to daily vitamin D intake during pregnancy and the seasonal distribution of deliveries showed that the mothers in the control group had higher rates of daily vitamin D intake and spring-summer deliveries, which corresponded well to significantly lower levels of maternal and newborn vitamin D levels in the sepsis group.

### Limitations of this study

The low socio-economic level of the participants and the exclusion of premature infants might have influenced maternal and newborn vitamin D levels. In addition, the number of patients in the LOS group was less than the EOS group.

In conclusion, despite the presence of few studies, the findings suggest that there may be an association between vitamin D deficiency and neonatal sepsis. In countries where vitamin D deficiency is common among pregnant women, the initiation of and effective implementation of vitamin D supplementation programs would be of particular value in reducing the health problems associated with vitamin D deficiency encountered not only among pregnant women but also among newborns. Yet, the level of vitamin D required for adequate immune function has yet to be clarified.

### Authors Contribution

**AAO:** Designed and did data collection, writing & editing of manuscript.

**YC:** Did data collection statistical analysis and manuscript writing.
